# Micropenis

**DOI:** 10.1100/tsw.2011.135

**Published:** 2011-07-28

**Authors:** Jeremy Wiygul, Lane S. Palmer

**Affiliations:** Division of Pediatric Urology, Cohen Children's Medical Center of New York of the North Shore-Long Island Jewish Health System, Long Island, NY, USA

**Keywords:** penis, micropenis, child, testosterone, surgery

## Abstract

Micropenis is part of a larger group of conditions broadly known as inconspicuous penis; however, it is fundamentally different from the other diagnoses in this group, such as webbed penis and buried penis, in that the underlying problem is the size of the penis itself, not with the surrounding and overlying skin. This condition is usually the result of a defect in the hypothalamic-pituitary-gonadal axis, although iatrogenic causes are identified infrequently. Management revolves around testosterone (direct administration or encouraging the patient's body to make its own), and long-term results with respect to increase in penile length are promising. Reconstructive surgery is based on the use of a vascular pedicle free flap and is reserved for patients who fail to respond to hormonal treatment. Although substantial long-term data are lacking, adult patients with micropenis appear to report dissatisfaction with penile appearance, but the majority appear to have adequate sexual function.

## INTRODUCTION

Micropenis is an oft-misapplied diagnosis in medicine that, when used inaccurately, can cause considerable parental anxiety and can take a good amount of effort to overcome. The term refers to a specific disorder that has a specific set of causative factors and a different set of treatment modalities than the broader term of inconspicuous penis or, apparently, small penis, in lieu of which it is generally used. It would therefore behoove us to understand fully the meaning and genesis of micropenis to better differentiate it from the other types of inconspicuous penis.

The definition of micropenis hinges on the idea of stretched penile length, or SPL. SPL was first introduced by Schonfeld and Beebe[1] in their seminal work determining standard penile length according to age. Micropenis is defined as a SPL 2.5 standard deviations less than the mean for age group[2] without the presence of any other penile anomalies, such as hypospadias. Thus, for newborn term infants, a SPL of 1.9 cm or less is micropenis (see Table 1).

**TABLE 1**
**Normal SPL**

| Age | Mean ± SD | Mean - 2.5SD |
|---|---|---|
| Newborn, 30-week gestation | 2.5 ± 0.4 | 1.5 |
| Newborn, 34-week gestation | 3.0 ± 0.4 | 2.0 |
| 0–5 months | 3.9 ± 0.8 | 1.9 |
| 6–12 months | 4.3 ± 0.8 | 2.3 |
| 1–2 years | 4.7 ± 0.8 | 2.6 |
| 2–3 years | 5.1 ± 0.9 | 2.9 |
| 3–4 years | 5.5 ± 0.9 | 3.3 |
| 4–5 years | 5.7 ± 0.9 | 3.5 |
| 5–6 years | 6.0 ± 0.9 | 3.8 |
| 6–7 years | 6.1 ± 0.9 | 3.9 |
| 7–8 years | 6.2 ± 1.0 | 3.7 |
| 8–9 years | 6.3 ± 1.0 | 3.8 |
| 9–10 years | 6.3 ± 1.0 | 3.8 |
| 10–11 years | 6.4 ± 1.1 | 3.7 |
| Adult | 13.3 ± 1.6 | 9.3 |

## EMBRYOLOGY

As with many genital disorders, an understanding of the relevant embryology allows a better understanding of the condition itself. Beginning at 8 weeks of gestation, maternal chorionic gonadotropins from the placenta begin to stimulate testosterone production from the fetal Leydig cells. Under the influence of dihydrotestosterone, a conversion product of testosterone, penile differentiation occurs. The genital tubercle differentiates into the glans penis, the genital folds become the shaft of the penis, and the genital swellings migrate to the midline to become the scrotum. Penile differentiation is complete by 12 weeks of gestation. During the second and third trimester, growth of the penis is accomplished through fetal androgens, which are produced under stimulation by fetal pituitary gonadotropin. There is a marked increased in penile size over that time period, with the penis growing almost 20 mm from weeks 16 to 38[3,4]. Therefore, true micropenis must result from a hormonal abnormality that occurs after 12 weeks of gestation. Studies of genital skin fibroblasts in patients with micropenis have shown normal androgen production and action after administration of gonadotropins, as well as appropriate receptor activity, which reinforces the central role the hypothalamic-pituitary axis played in the genesis of micropenis[5].

## ETIOLOGY

True micropenis is a result of a hormonal abnormality occurring after 12 weeks of gestation. The causes of this condition can be divided into three broad groups: hypogonadotropic hypogonadism (pituitary/hypothalamic failure), hypergonadotropic hypogonadism (primary testicular failure), and idiopathic. These represent the most common etiologies of micropenis[6,7,8]. Table 2 highlights the different etiologies.

**TABLE 2**
**Etiologies**

I. Deficient testosterone secretion
   A. Hypogonadotropic hypogonadism
      1. Isolated, including Kallmann's syndrome
      2. Associated with other pituitary hormone deficiencies
      3. Prader-Willi syndrome
      4. Laurence-Moon syndrome
      5. Bardet-Biedl syndrome
      6. Rud's syndrome
   B. Primary hypogonadism
      1. Anorchia
      2. Klinefelter's and poly-X syndromes
      3. Gonadal dysgenesis (incomplete form)
      4. Luteinizing hormone receptor defects (incomplete forms)
      5. Genetic defects in testosterone steroidogenesis (incomplete forms)
      6. Noonan's syndrome
      7. Trisomy 21
      8. Robinow's syndrome
      9. Bardet-Biedl syndrome
      10. Laurence-Moon syndrome
II. Defects in testosterone action
   A. Growth hormone/insulin-like growth factor-I deficiency
   B. Androgen receptor defects (incomplete forms)
   C. 5-α reductase deficiency (incomplete forms)
   D. Fetal hydantoin syndrome
III. Developmental anomalies
   A. Aphallia
   B. Cloacal exstrophy
IV. Idiopathic
V. Associated with other congenital malformations

Typically, when due to hypogonadotropic hypogonadism, micropenis is associated with conditions such as Kallman's syndrome (hypogonadotropic hypogonadism and anosmia) and Prader-Willi syndrome (hyperphagia, mental retardation, short stature, hypotonia, and hypogonadism) (see Table 2). Hypergonadotropic hypogonadism, or primary testicular failure, can be due to gonadal dysgenesis, or may be associated with Robinow's syndrome[6] as well as poly-X syndromes, such as XXY (Klinefelter's syndrome), gene translocations, and trisomies of chromosome 8, 13, and 18[2]. Problems with testosterone action, such as 5-α reductase deficiency, can present as micropenis in its incomplete form, although hypospadias is a much more common result[6]. Finally, idiopathic causes of micropenis are associated with an empirically normal hypothalamus-pituitary-testicular axis[6].

## DIAGNOSIS

As stated previously, to make an accurate diagnosis of micropenis, the examining clinician must have a clear understanding of the definition of micropenis as well as how to measure the penis. This is to exclude confounding diagnoses, such as webbed penis and hidden penis. SPL is measured from the point where the penis meets the pubic bone to the distal tip of the penis, which is put on maximal stretch (Fig. 1). Care must be taken to compress any suprapubic fat pad, prevalent in infants and most likely the major cause of misdiagnosis of micropenis in this age group. The micropenis typically has a normal circumference-to-length ratio, although, rarely, severely hypoplastic corpora cavernosa will be seen[8]. Frequently, micropenis is associated with cryptorchidism and small-volume testicles, as well as a hypoplastic scrotum,

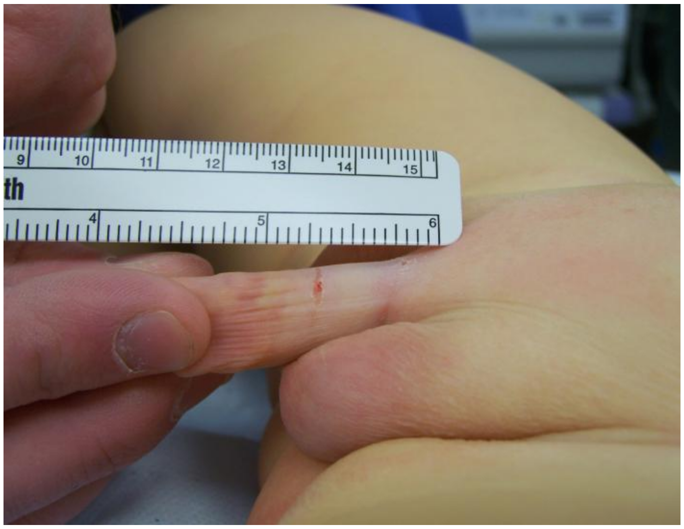

**FIGURE 1.** SPL is measured from the pubis to the tip of the stretched penis.

most likely due to the same causative factors that are responsible for the micropenis[8]. Other characteristics, such as delayed puberty in older children, suggestive of hypogonadotropic hypogonadism, should be noted to aid in diagnosis.

Once micropenis is confirmed through physical exam, consultation with the endocrinology service should be obtained to help determine the cause of micropenis as well as to rule out possible life-threatening associated abnormalities. Specifically, hypogonadotropic hypogonadism is commonly associated with growth hormone (GH) deficiency and/or adrenocorticotropic hormone (ACTH) deficiency, putting the infant at high risk for death due to hypoglycemia or cortisol deficiency[9]. Plasma cortisol, serum electrolytes, and plasma glucose may be obtained in this setting to rule out acute problems. The endocrinologic evaluation can also isolate the cause of micropenis to its level in the hypothalamic-pituitary-testicular axis[9]. Specifically, prolactin (PRL) levels help to isolate the defect to the hypothalamus (high PRL) vs. the pituitary (low PRL)[9,10]. In addition, plasma GH, thyroid stimulating hormone (TSH), and ACTH can all be used to isolate the location of dysfunction[9]. Interestingly, it may be difficult to make the diagnosis of hypogonadotrophic hypogonadism in the prepubertal patient with micropenis if they are past infancy, as there is a quiescent phase of the pituitary that sees levels of follicle-stimulating hormone (FSH) and luteinizing hormone (LH) drop precipitously[9].

In parallel with an evaluation of central endocrine function, testicular function may also be assessed through serum testosterone levels before and after administration of human chorionic gonadotropin (hCG). No rise in postadministration testosterone and rises in LH and FSH are consistent with testicular failure or absence, although this can also occasionally be seen in those patients with Kallman syndrome and undescended testicles[11]. Antimüllerian substance (AMH), also known as müllerian-inhibiting substance (MIS), and inhibin B, which are produced by functioning Sertoli cells, can also be used to determine the presence of functional testicular tissue[9,12]. Low AMH coupled with a normal inhibin B is indicative of the rare, persistent müllerian duct syndrome, which is the result of a defect in the gene that encodes AMH[9].

Magnetic resonance imaging (MRI) may be used to identify midline structural defects, such as pituitary stalk dysplasia syndrome, central diabetes insipidus (indicated by a lack of a posterior pituitary bright spot), and pituitary aplasia[9,13,14]. Specifically, findings such as a small anterior pituitary gland, attenuated or absent pituitary stalk, and ectopic posterior pituitary are all suggestive of hypopituitarism and thus can help to facilitate identification of the etiology[14,15]. Finally, some authors advocate obtaining a karyotype[11], although this recommendation is not universal.

## TREATMENT

Treatment of micropenis should focus on penile size sufficient for the patient to have an appropriate body image, normal sexual function, and standing micturition. Inability to bring the penis fully to the mean measurement for age does not imply failure. Primary treatment of micropenis revolves around exogenous testosterone administration to increase the length of the penis so that it may be considered within a range of normal. Most authors endorse 25 mg of intramuscular testosterone in infancy, typically in its ethanate formulation to promote longer action, once a month for 3 months[8,9,11], followed by further courses at higher dosages at the start of pubarche[16]. Good responses are typically seen, with increases of over 100% in penile length over the course of initial treatment to be expected[16,17]. However, if the response is not deemed satisfactory, repeat administrations over short time periods may be performed without significant concern about early maturation of bony growth plates and subsequent reduction in stature[9,16]. Transdermal delivery of both testosterone and dihydrotestosterone have also been reported[18,19], with the application of dihydrotestosterone resulting in increases in penile length of over 150% during the treatment period[18], although others have found no such results in head-to-head comparisons with respect to testosterone[20]. There has also been two reports of administering LH and FSH to an infant with hypogonadotrophic hypogonadism and micropenis; however, although there was a significant increase in testicular volume over the treatment period in both studies, minimal increase in penile size was noted in one study[21] and was seen in one out of two patients in another[22]. This is in keeping with the gonadotropin releasing hormone (GnRH) surge seen immediately after birth in normal infants[9], with accompanying increases in Sertoli cell populations[23].

In light of all this, there have also been concerns voiced about the administration of testosterone to prepubertal patients and the impact on their ultimate penile length. Current long-term data regarding patients treated in childhood with exogenous testosterone have shown no reduction in adult penile length[24]. It should be emphasized, however, that the impact of testosterone treatment in childhood on masculinization during puberty is still not fully described and better long-term data are needed to fully understand the effects of treatment.

If endocrine treatment does not accomplish a satisfactory result, surgical therapy can offer an alternative in the management of micropenis. Early writers on micropenis endorsed sex reassignment[25,26], especially if there was evidence of lack of testicular tissue[25]. However, more recently, the lack of data regarding the long-term psychological impact of gender reassignment in pediatric patients[27], coupled with some high-profile cases of patients who were sex reassigned in childhood having spectacularly bad outcomes, has called into question the wisdom of this approach. While some data do seem to promote the idea that most sex-reassigned patients are comfortable with their assigned sex, regardless of chromosomal sex[28,29], and others have found mixed results[30], still other researchers have found significant psychosexual problems in adult patients who had undergone sex reassignment as children[31]. Due to the relatively small number of patients in whom the discussion of sex reassignment in infancy is indicated, most studies of outcomes in this group include a range of etiologies, most of which entail a markedly different fetal and infantile hormonal milieu than those patients with micropenis[28,29,30,31]. Therefore, extrapolation to the patients with micropenis who underwent sex reassignment is fraught. In light of this, sex reassignment with creation of female genitalia for patients with this condition should be undertaken with extreme caution and should only be done by those with a large amount of experience.

Reconstructive surgery has a long history in the treatment of micropenis, with Frank Hinman publishing his initial results of reconstruction of patients with micropenis in the early 1970s[32]. Further advances in penile reconstruction began in the 1980s with the description of a faciocutaneous neophallus based on the radial artery of the forearm[33]. Other techniques for phallic reconstruction include the sensate osteocutaneous fibula flap, the free scapular flap, the suprapubic abdominal wall flap, and the vertical rectus abdominis flap, although the radial forearm free flap remains the most popular in terms of phallic reconstruction[34]. Cosmetic and functional results are acceptable, especially when a prosthesis is implanted after reconstruction[35]; however, despite being used in select patients, the complication rate remains dauntingly high, even in the most experienced of hands[35,36]. While most donor site complications are minimized with increasing experience, complications with the flap (and urethral anastomosis, if the patient undergoes one) continue to challenge reconstructive surgeons, regardless of experience. One large study showed a flap revision rate of 12%[35], while another cohort had 53 patients undergoing a mean of six operations to achieve a lasting neophallus[36]. Corporal augmentation procedures have also been described in the setting of micropenis, with acceptable short-term results, although no long-term data exist[37].

Most likely the biggest problem associated with the management of micropenis is the lack of knowledge in terms of long-term outcome. With regards to long-term sexual function and gender identity, Reilly and Woodhouse[38] studied a group of 20 patients with micropenis, unresponsive to hormonal therapy as children, raised as males, ranging in age from 10 to 43 years of age. They found all patients reporting male gender identity, erections, and orgasm. In addition, nine of the 12 adult patients were sexually active, although half also experienced teasing due to genital appearance[38]. In a group of 22 adult micropenis patients raised as males, Lee and Houk reported similar findings[39]. Satisfaction with genital appearance remains an issue, however[40]. Overall, it may safely be said that current evidence points to normal gender identity and sexual function in the majority of patients with micropenis raised as males, even if their micropenis is not corrected.

## SUMMARY

Micropenis is part of a larger group of conditions broadly known as inconspicuous penis; however, it is fundamentally different from the other diagnoses in this group, such as webbed penis and buried penis, in that the underlying problem is the size of the penis itself, not with the surrounding and overlying skin. This condition is usually the result of a defect in the hypothalamic-pituitary-gonadal axis, although iatrogenic causes are identified infrequently. Since micropenis can be the first manifestation identified of a broader endocrinologic problem, a pediatric endocrinologist should be consulted once the diagnosis of micropenis is made. Treatment of micropenis revolves around testosterone, either through direct administration or encouraging the patient’s body to make its own, and long-term results in terms of increased penile length are promising. Reconstructive surgery comes with a variety of options, all based on the principle of a vascular pedicle free flap, and is reserved for those patients not responding to hormonal treatment. Patients with micropenis in adulthood do report dissatisfaction with the appearance of their penis, but the majority appears to have adequate sexual function. It should be stated, however, that long-term robust data are still lacking in this crucial area.
